# *Panax notoginseng saponins* promote endothelial progenitor cell angiogenesis via the Wnt/β-catenin pathway

**DOI:** 10.1186/s12906-021-03219-z

**Published:** 2021-02-08

**Authors:** Peiqi Zhu, Weidong Jiang, Shixi He, Tao Zhang, Fengchun Liao, Di Liu, Xiaoning An, Xuanping Huang, Nuo Zhou

**Affiliations:** 1 Guangxi Medical University, Nanning, 530021 People’s Republic of China; 2 Department of Oral and Maxillofacial Surgery, Hospital of Stomatology, Guangxi Medical University, Nanning, 530021 People’s Republic of China; 3 Guangxi Key Laboratory of Oral and Maxillofacial Rehabilitation and Reconstruction; Guangxi Key Laboratory of Oral and Maxillofacial Surgery Disease Treatment, Guangxi Clinical Research Center for Craniofacial Deformity, Nanning, 530021 People’s Republic of China

**Keywords:** Panax notoginseng saponins, Endothelial progenitor cells, Wnt/β-catenin signaling, Angiogenesis

## Abstract

**Background:**

Distraction osteogenesis (DO) is an effective treatment in craniomaxillofacial surgery. However, the issue of sufficient blood supply at the regeneration tissue has limited its wide application. *Panax notoginseng saponins* (PNS) is a Traditional Chinese Medicine that is commonly used to treat a range of angiogenic diseases. However, the mechanisms whereby PNS alters angiogenesis in endothelial progenitor cells (EPCs) have yet to be clarified.

**Methods:**

EPCs were identified by immunofluorescence, confirmed by their uptake of fluorescently labeled Dil-ac-LDL and FITC-UEA-1. EPCs were treated with different concentrations of PNS, and the effects of PNS on cell proliferation were measured on the optimal concentration of PNS determined. The effects of PNS on angiogenesis and migration, angiogenic cytokines mRNA expression and the proteins of the Wnt pathway were investigated. Then knocked down β-catenin in EPCs and treated with the optimum concentrational PNS, their angiogenic potential was evaluated in tube formation and migration assays. In addition, the expression of cytokines associated with angiogenesis and Wnt/β-catenin was then assessed via WB and RT-qPCR.

**Results:**

We were able to determine the optimal concentration of PNS in the promotion of cell proliferation, tube formation, and migration to be 6.25 mg/L. PNS treatment increased the mRNA levels of VEGF, bFGF, VE-Cadherin, WNT3a, LRP5, β-catenin, and TCF4. After knocked down β-catenin expression, we found that PNS could sufficient to partially reverse the suppression of EPC angiogenesis.

**Conclusions:**

Overall, 6.25 mg/L PNS can promote EPC angiogenesis via Wnt/β-catenin signaling pathway activation.

## Background

Reconstruction of bone deformities and defects caused by congenital malformation, injury, or tumorectomy represent significant clinical challenges in oral and maxillofacial surgery [1]. In contract to conventional treatments, such as autologous bone grafts, distraction osteogenesis (DO) is unique in its ability to induce new bone formation between two osteotomized bone segments separated by gradual distraction [2], and has become a powerful tool for reconstructing mandibular defects. However, DO requires longer times for bone consolidation, a drawback when time is limited, and which has impeded its widespread use [3]. Therefore, novel and effective approaches are urgently needed to accelerate bone regeneration during the consolidation process and thus to improve the clinical efficacy of DO.

The application of Traditional Chinese medicine (TCM) to bone regeneration and angiogenesis has stimulated considerable interest in recent years. *Panax notoginseng saponins* (PNS) is a TCM compound that is isolated from Panax notoginseng (Burk.) F. H. Chen [4]. Historically, PNS has been used to relieve pain, to eliminate blood stasis, and to reduce bleeding as proscribed in the Compendium of Materia Medica simply saying [5]. In addition, PNS mitigates estrogen deficiency-induced deterioration of the trabecular microarchitecture and suppresses marrow adipogenesis [6]. Moreover, our previously research has domenstrated that PNS promotes osteogenesis during DO [7]. There is also evidence that PNS can promote angiogenesis and thereby drive rapid wound healing [8], with this pro-angiogenic activity being at least partially associated with the ability of PNS to enhance the expression of VEGF [9]. However, the molecular mechanisms whereby PNS can influence angiogenic processes remain to be fully clarified.

Recently, endothelial progenitor cells (EPCs), isolated from the peripheral blood, cord blood, and the bone marrow [10], have been shown to play essential roles in the vascularization of ischemic tissues through a multi-stage process wherein these cells migrate to these tissues and therein orchestrate angiogenesis [11]. Bone regeneration is closely coupled with angiogenesis. Observations on the primary mineralizing front in DO suggest that there is tight spatial coordination between the areas of neovascularization and new bone formation [12]. In the process of DO, the distraction zone becomes relatively ischemic during activation and it has been found that EPCs accumulate at this site during the activation phase and remain there during the consolidation phase [13]. Meanwhile, signals from the DO regeneration site promote the mobilization of EPCs from the bone marrow into the peripheral circulation, accelerating bone healing by angiogenesis–osteogenesis coupling [14, 15]. However, the role of PNS in the promotion of EPC-mediated-angiogenesis during DO remains unknown. In previous study, we demonstrated that treatment with PNS could increase both new bone formation [7] and EPC promotion of vascularization during DO [16]. Based on these findings, we hypothesized that PNS could promote the EPC-mediated angiogenesis during DO. And in this study, we aimed to explore the underlying mechanism of PNS on EPC angiogenesis.

## Methods

### Canine EPC isolation and culture

Two-week-old male Beagle dogs were purchased from the Experimental Animal Center of Guangxi Medical University (Nanning, China). All animal experimental procedure was approved by the Animal Care and Use Committee of Guangxi Medical University. The isolation procedure was performed as described in previous studies [17]. Mononuclear cells were isolated from the bone marrow by density gradient centrifugation with Percoll cell separation solution (Solarbio, China), plated in T25 cell culture flasks coated with fibronectin (Sigma, USA) overnight and then cultured in complete endothelial growth medium-2 (EGM-2; Lonza, USA) at 37 °C in a 5%CO_2_ humidified incubator. Cells were cultured until 80–90% confluent, at which time they were passaged. Following two such passages, cells were utilized for downstream experiments.

### Dil-ac-LDL uptake and FITC-UEA-1 binding of EPCs

EPCs were washed with phosphate-buffered saline (PBS) three times, and incubated in medium containing 20 μg/ml Dil-ac-LDL (YEASEN Biotech, China) for 4 h at 37 °C, 5% CO_2_. Cells were fixed with 4% paraformaldehyde and incubated for 1 h with 10 μg/ml FITC-UEA-1 (Invitrogen, USA) at room temperature. Nuclei were labeled with DAPI (C0065, Solarbio, China). The incorporation of Dil-Ac-LDL and binding of FITC-UEA-1 were assessed and photos were taken in an Intelligent Full-Automatic Fluorescence Microscopy Imaging System (Invitrogen™ EVOS™ FL Auto 2, Thermo Scientific, USA).

### Immunofluorescence analyses

Immunofluorescent staining for CD34, CD133, VEGFR2, and β-catenin was conducted using EPCs after two passages. Briefly, these cells were plated in 12-well plates (1× 10^4^/well) until 80% confluent, at which time they were fixed for 15 min using 4% paraformaldehyde (PFA), then permeabilized with 0.1% Triton X-100 for 20 min, and blocked for 30 min using 10% goat serum. These cells were then incubated overnight with mouse monoclonal anti-CD34 (12,034,042,1:50; eBioscience, USA), goat polyclonal anti-VEGFR2 (ab10972, 1:500; Abcam), rat monoclonal anti-CD133 (12,133,182, 1:100; eBioscience, USA), and/or mouse monoclonal anti-Beta-Catenin (138,400, 1:500; ThermoFisher) at 4 °C. Samples were then washed, probed for 1 h with secondary antibodies at 37 °C, and Nuclei were labeled with DAPI (C0065, Solarbio, China). Samples were then combined with 100 μl of anti-fluorescence attenuating mount, after which they were evaluated with an Intelligent Full-Automatic Fluorescence Microscopy Imaging System (Invitrogen™ EVOS™ FL Auto 2, Thermo Scientific, USA).

### Cell proliferation assay

Cell proliferation was assessed by assay with the Cell Counting Kit-8 (CCK-8, Dojindo, Japan). To determine the optimal concentrations of PNS for the subsequent experiments, EBM containing different concentrations of PNS was used. The EPCs were seeded in 96-well plates at a density of 3× 10^3^ cells/well in triplicate. After cell attachment, the culture medium was replaced by the medium supplemented with various concentrations of PNS. The 10 μL of CCK-8 solution was added to measure proliferation after 24 h, 48 h, 72 h and 96 h. Absorbance was measured using a microplate reader at 450 nm.

### β-Catenin knockdown in EPCs

To knock down β-catenin, we purchased lentiviruses encoding short hairpin RNAs (shRNAs) specific for this gene or control shRNAs from GeneChem (China). The following three candidate shRNAs were used in these analyses: GCACCATGCAGAATACAAA (shRNA-β-catenin1), GGACCTACACTTACGAGAA (shRNA-β-catenin2), and GCTGCTTTATTCTCCCATT (shRNA-β-catenin3). Cells were transduced with these lentiviruses under appropriate conditions, after which β-catenin knockdown was confirmed via western blotting and RT-PCR, with the shRNA that achieved maximal knockdown being used for downstream studies.

### Cellular treatment

The impact of PNS on EPC angiogenesis was assessed by treating cells with a range of different conditions. Control (CTRL) cells were untreated and were not lentivirally transduced, while negative control (NC) EPCs were untreated and were transduced with a lentivirus encoding a control scrambled shRNA. In addition, cells in the shRNA-β-catenin group were transduced with an shRNA-β-catenin lentivirus, while cells in the CTRL + PNS group were treated with 6.25 mg/L PNS, those in the NC + PNS group were treated with 6.25 mg/L and were transduced with a control lentivirus, and those in the shRNA-β-catenin + PNS group were treated with 6.25 mg/L and were transduced with an shRNA-β-catenin lentivirus. To prevent any confounding results associated with the supplements in the EGM-2 media, cells were instead grown in basal EBM media containing 10% FBS (Gibco Co, USA) following passage. PNS treatment was conducted for 72 h in all appropriate groups.

### RT-qPCR

Trizol (Invitrogen, CA, USA) was used to extract cellular RNA, which was then used to generate cDNA with a RevertAid First Strand cDNA Synthesis Kit (Invitrogen) based on provided instructions. All primers were synthesized by Sangon Biological Engineering Technology & Services Co., Ltd. (Shanghai, China). A QuantStudio-5 system (Applied Biosystems) was used for RT-qPCR reactions with the following thermocycler settings: 50 °C for 2 min; 95 °C for 2 min; 40 cycles of 95 °C for 15 s, 60 °C for 1 min; 72 °C for 1 min. Each reaction well contained 10 μl of 2×PowerUp SYBR Green Master Mix (Invitrogen), 2 μl of each primer (800 nM),2 μl of cDNA (10 ng/μl), and 4 μl ddH2O. The 2^-△△Ct^ approach was used to assess relative gene expression, with GAPDH being used as a normalization control. The primer sequences are listed in Table 1.

**Table 1:** Primers sequences used in the RT-qPCR

Gene	Forward primer	Reverse Primer
WNT3a	5′- TGCCAGAATCGGTCACGCG-3’	5′-CACGTTGTTGGTGCGCTGC − 3’
WNT5a	5′-TTAAGGCCAGGAGTTGCTTTG − 3’	5′-CTGACATCTGAACAGGGTTATTCAT − 3’
LRP5	5′-CCTCCGCGCTAGACTTTGAT-3’	5′-AAGCTCGGCTGATGGTCTTC-3’
β-catenin	5′- GGAATGGCTACCCAAGCTGA − 3’	5′- AAGACTGTTGCTGCCAGTGA − 3’
TCF4	5′- GCCATCTTCAGTTTATGCTCC-3’	5′-TGATGGCCATCCTGCATGAAG − 3’
VEGF-A	5′- TCCACCATGCCAAGTGGT − 3’	5′- CCATGAACTTCACCACTTCG − 3’
bFGF	5′- AGAGAGCGTTGTGTCCATC − 3’	5′- GCCCAGTTCGTTTCAGTGC − 3’
VE-cadherin	5′- ACACAGCCAACATCACCGTCAAG-3’	5′- CCGCCATCTCCTCGCAGTAGG-3’
GAPDH	5′- ATTCCACGGCACAGTCAAGG-3’	5′-ACATACTCAGCACCAGCATC-3’

### Cellular protein extract preparation

EPCs were harvested in PBS and spun for 5 min at 12,000×g at 4 °C. Total protein extracts and nuclear protein extracts were then prepared from these samples using RIPA lysis buffer and a Nuclear and Cytoplasmic Protein Extraction kit (Beyotime, Jiangsu, China), respectively. In both cases, 1 mM PMSF (Beyotime) was added to solutions, and an Enhanced BCA kit (Beyotime) was used to quantify protein levels in isolated samples.

### Western blotting

Protein samples isolated as above were used for standard Western blotting experiments. Briefly, 12% SDS-PAGE was used to separate equal protein samples (30 μg each), which were then transferred to PVDF membranes (Millipore, MA, USA). Blots were next blocked for 1 h using 5% non-fat milk in TBST, prior to incubation overnight with antibodies specific for Dishevelled (DVL) (Invitrogen), β-catenin (ThermoFisher), glycogen synthase kinase-3b (GSK-3β) (BIOSS, Beijing, China), (phospho-S9)-GSK-3b (BIOSS), VEGF-A (ThermoFisher), bFGF (AVIVA SYSTEMS BIOLOGY, USA), VE-Cadherin (AVIVA SYSTEMS BIOLOGY), histone H3 (Proteintech, China), and β-actin (Beyotime) (all 1:1000 in TBST) at 4 °C. Blots were then washed thrice using TBST (5 min/wash) prior to an additional 1 h room temperature incubation with biotin-conjugated secondary antibodies (1:1000 in TBST) (Beyotime). A BeyoECL Plus kit (Beyotime Bio) was then used to detect all protein samples based on provided directions, with ImageJ (https://imagej.nih.gov/ij/) being used for densitometric analyses. All experiments were repeated a minimum of three times.

### In vitro capillary tube formation assay in Matrigel

EPC tube formation was assessed in Matrigel as previously described [18]. One day before this assay, Matrigel (Corning Co. Ltd., USA) was melted into a liquid state at 4 °C overnight. The following morning, a μ-Slide Angiogenesis plate was coated using 10 μl of this Matrigel at 4 °C for 30 min. Plates were then transferred to 37 °C in a 5% CO_2_ humidified incubator for 30 min to facilitate Matrigel solidification. Next, 1.5 × 10^4^ EPCs that had been stained using Calcein (ThermoFisher Co. Ltd., USA) and cells were returned to the incubator for 6 h. Cells were then imaged with an inverted microscope (100 ×) to assess tube formation. For each sample, at least five micrographs were taken at different positions. The ability of EPCs to form tube-like structures was assessed by counting the number of network circles in each image.

### Migration assay

Cell migration was measured in culture inserts containing microporous membranes (8.0 μm, Corning, NY, USA). Briefly, 5× 10^3^ cells/well suspended in EBM were seeded in the upper chamber of a modified Boyden chamber (Corning Costar, Corning, NY, USA), and PNS was added to the lower chamber to induce EPC migration. After 72 h incubation at 37 °C and 5% CO_2_, the microporous membranes were washed with PBS, fixed in 4% paraformaldehyde for 15 min, and stained with 0.5% crystal violet solution. Non-migrating cells on the top side were removed with cotton-tipped swabs and the number of migrated cells was counted manually in five random microscopic fields (100 ×) in each insert.

### Statistical analysis

SPSS 23.0 was used for all statistical analyses. Data were compared via Student’s t-tests and one-way ANOVAs, as appropriate. All data were presented as means ± standard deviation, and *p* < 0.05 was the significance threshold. *p-values < 0.05, **p < 0.01, ***p < 0.001, #*p* < 0.05, or ##*p* < 0.01 were considered as statistically significant. All experiments were repeated at least three times to confirm reproducibility.

## Results

### EPC isolation, cultivation, and identification

**Fig. 1:**
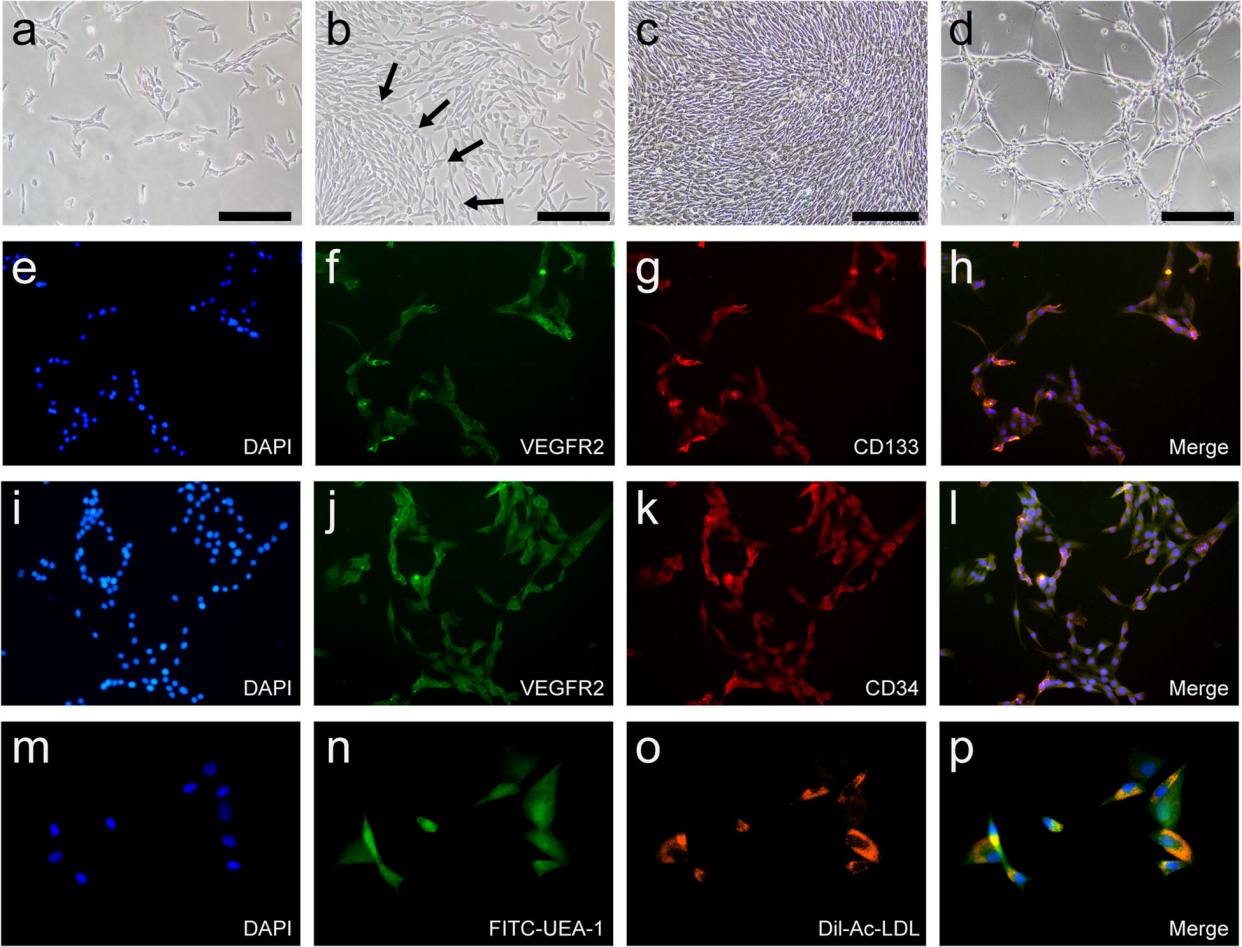
Isolation, cultivation, and identification of EPCs. (**a**-**d**) Sequential changes of EPCs. **a** A small amount of adherent cells appeared 3–4 days after seeding (100×). **b** EPCs colonies formed 7 days after seeding (100×, arrows showed a representative colony). **c** EPCs colonies grew to confluence and exhibited a cobblestone-like cell monolayer 10 days after seeding (100×). **d** EPCs formed tube-like structures on Matrigel in vitro (100×). **e**-**l** Fluorescent microscopy was used to characterize cell surface marker expression on canine EPCs from passage 2. VEGFR2 (**f**, **j**), CD133(**g**), CD34 (**k**) expression being assessed. **n** EPC uptake FITC-UEA-I (green, 400×); **o** EPC uptake of Dil-Ac-LDL (red, 400×)

EPCs were isolated from canine bone marrow by density gradient centrifugation. As shown in Fig. 1a, small numbers of adherent cells appeared after 3–4 days’ culture. Colonies were observed seven days after seeding (Fig. 1b). By day 10, the colonies had fused with one another, resulting in a larger cell monolayer with a cobblestone-like morphology (Fig. 1c), and gradually grew outward. Some EPCs formed tube-like structures on Matrigel during cultivation (Fig. 1d); this is a functional property of EPCs. Furthermore, the identity of the EPCs was confirmed by examining the expression of the endothelial cell surface antigen VEGFR-2 (Fig. 1f, j) and the progenitor cell surface antigens CD133 (Fig. 1g) and CD34 (Fig. 1k), consistent with the results of previous studies characterizing these cells [10, 19]. As shown in Fig. 1, almost all of the EPCs expressed these surface antigens. EPCs that had incorporated Dil-Ac-LDL emitted red fluorescence, while green emission was seen with those incorporating FITC-UEA-1 (Fig. 1m-p). These functions are characteristic functions of EPCs.

### PNS treatment promotes EPC prolifieration, tube formation, and migration in vitro

**Fig. 2:**
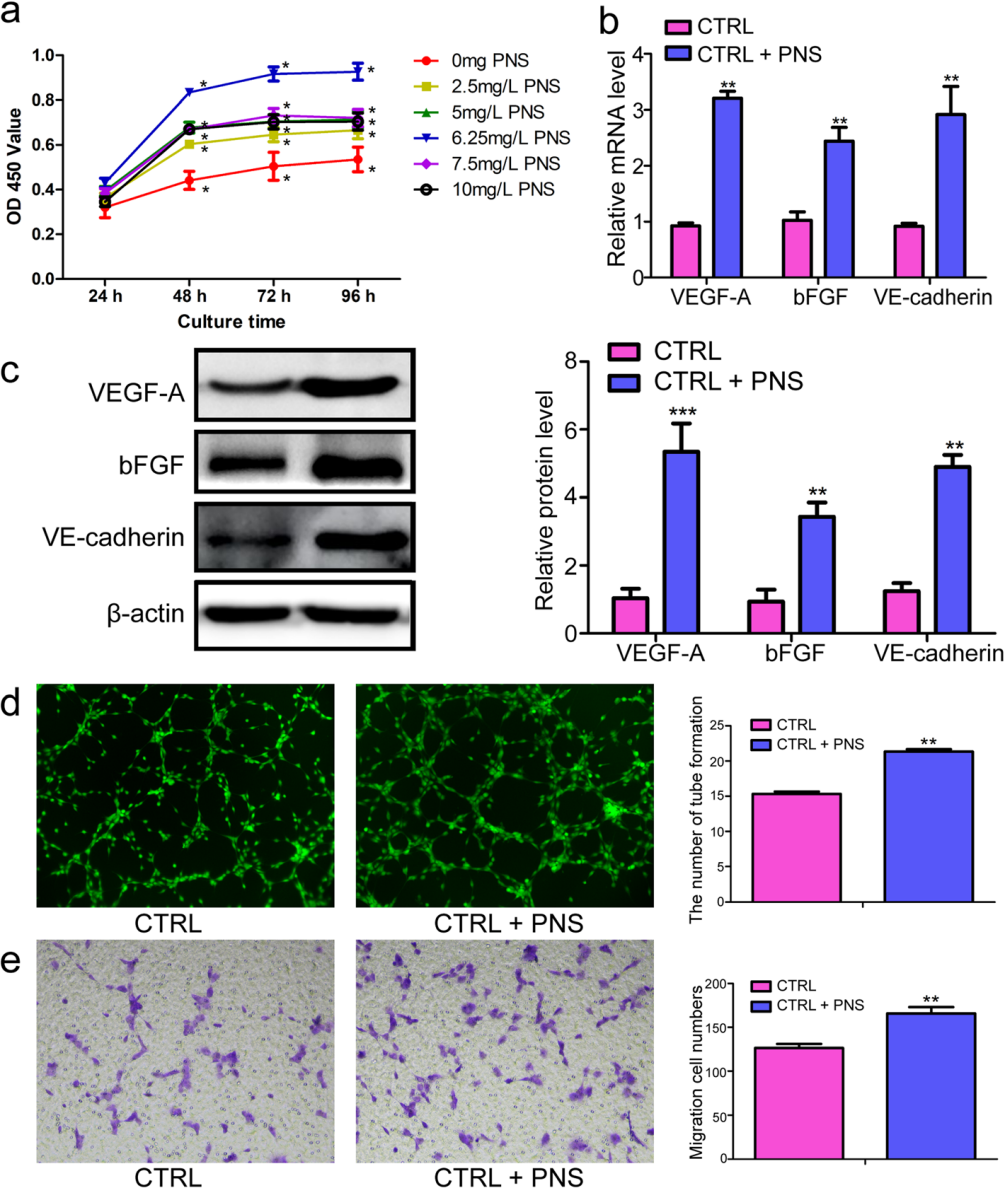
PNS treatment promote EPCs angiogenesis and detection the angiogenesis ability. **a** CCK8 assay for EPCs cultured in different concentrations of PNS at 24 h, 48 h, 72 h and 96 h. **b**-**c** qRT-PCR and WB analysis for the expression levels of VEGF-A, bFGF and VE-cadherin treated with EBM or PNS+EBM medium, respectively. **d**-**e** After PNS was used to treat EPCs, tube formation and migration was assessed. The tube length and the number of migrating cells were evaluated by counting 5 random fields at × 200 magnification. **compare to CTRL group, *p* < 0.01

The proliferation of EPCs was higher after treatment with different concentrations of PNS compared to control group at every time point. Notably, the results demonstrated optimal proliferation at the 6.25 mg/L dose (Fig. 2a). Consequently, 6.25 mg/L PNS was chosen as the appropriate concentration for further study. VEGF-A and bFGF are well-known to be key mediators of growth and angiogenesis [20] and are capable of promoting endothelial cell growth and migration, tubular structure formation, and EPC differentiation and maturation [21]. Meanwhile, we found that PNS treatment was associated with significant increases in both the mRNA and protein expression of VEGF-A, bFGF and VE-cadherin, as detected by qRT-PCR and WB (Fig. 2b, c). The tube formation assay is one measure of in vitro angiogenic function. To evaluate the impact of PNS on the ability of EPCs to form vascular-like structures, we utilized Matrigel as a basement membrane to facilitate the formation of these vascular structures, with the pro-angiogenic activity of PNS then being quantified by evaluating features of these networks such as branch point formation. Through this approach, we found that PNS-treated cells exhibited significantly more branch point formation than did untreated control EPCs (Fig. 2d, *p* < 0.05). After culturing for 72 h, the migration rate of EPCs exposed to PNS was significantly greater than that of the control group (Fig. 2e, *p* < 0.05). These data suggested that PNS promotes proliferation, migration, and angiogenesis of EPCs, functions that are essential for bone regeneration.

### The effect of PNS on EPCs is mediated by the Wnt/β-catenin signaling pathway

**Fig. 3:**
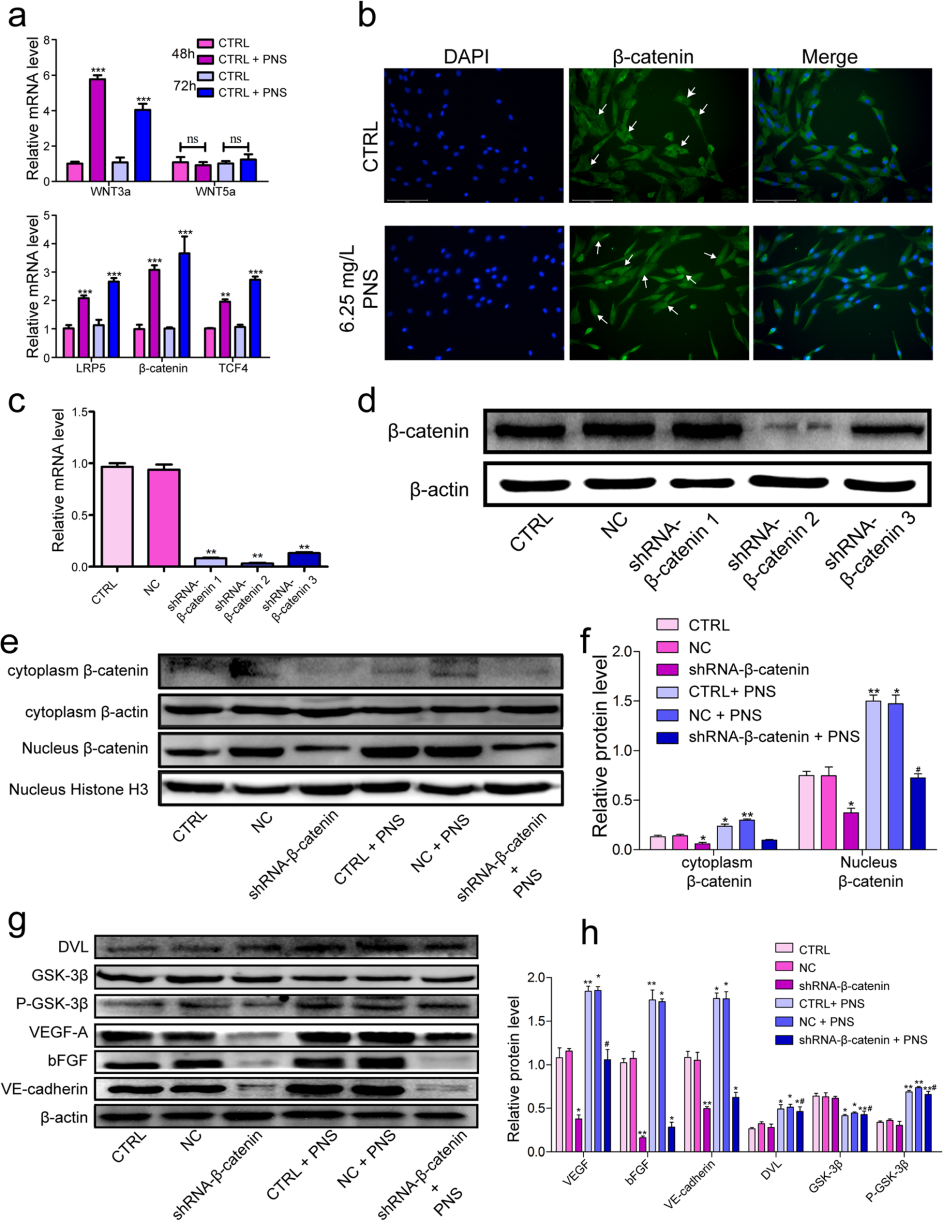
The Effect of PNS on EPCs is Mediated by the Wnt/β-catenin Signaling Pathway. **a** The mRNA expression of Wnt3a, Wnt5a, LRP5, β-catenin and TCF4 was detected by qRT-PCR. **b** Immunofluorescent microscopy was used to elucidate the cellular localization of β-catenin after treated with PNS. **c**, **d** The expression of β-catenin was assessed at the mRNA (**c**) and protein (**d**) levels in control cells, NC cells, and cells transduced with three different shRNA constructs targeting β-catenin. **p < 0.01 v.s. CTRL. **e**-**f** Effect of PNS treatment on nuclear enrichment of Wnt/β-catenin Pathway transcription factors. EPCs were exposed to increasing concentrations of PNS and incubated for 72 h. At the end of the incubation period, cytosolic and nuclear protein extracts were prepared and subjected to WB analysis. Protein levels of β-catenin were measured in nuclear and cytosolic protein fractions. * *p* < 0.01, ** *p* < 0.01 v.s. CTRL. **g**-**h** PNS promotes angiogenesis-related protein expression and Wnt/β-catenin signaling pathway activation. Western blot to evaluate the protein levels of VEGF-A, bFGF, VE-cadherin, DVL, GSK-3β and P-GSK-3β in indicated groups. The average levels were determined by ImageJ for three independent experiments. * *p* < 0.05, ** *p* < 0.01, ## *p* < 0.01 v.s. shRNA-β-catenin group

The Wnt signaling pathway have been implicated in a wide spectrum of biological phenomena such as vasculogenesis [22], new bone formation [23] and tissue regeneration [24]. Our study aimed to explore whether the canonical Wnt/β-catenin signaling pathway or non-canonical WNT pathway was involved in regulating the proliferation, angiogenesis, and migration of EPCs treated with PNS. To explore the potential mechanisms by which PNS regulates the biological processes of EPCs, we first assessed the mRNA levels of WNT3a and WNT5a in these cells treated with or without PNS via qRT-PCR. The results showed that PNS could strikingly increase WNT3a expression (Fig. 3a, *p* < 0.001). Meanwhile, the mRNA levels of LRP5, β-catenin and TCF4 were also elevated (Fig. 3a, *p* < 0.01). These findings indicated that the canonical Wnt signaling pathway may participate in this process. To further elucidate the cellular localization of β-catenin, immunofluorescence staining analysis was performed, which confirmed that the presence of β-catenin in EPCs. PNS clear promoted the expression of β-catenin, and it can be seen that β-catenin was largely transferred into the cell nucleus in the PNS groups, a significantly different situation from that in the control group where it barely entered the cell nucleus (Fig. 3b).

Wnt/β-catenin pathway activation leads to the cytoplasmic accumulation and subsequent nuclear translocation of β-catenin, which is then able to promote the transcription of a number of downstream target genes [25]. To illustrate the mechanism of PNS, we employed an shRNA transduction approach in order to stably knock down β-catenin in these cells. Subsequent RT-qPCR (Fig. 3c, *p* < 0.01) and WB (Fig. 3d) results revealed that this shRNA approach was sufficient to knock down β-catenin levels in these EPCs. Of the three tested shRNA constructs, the shRNA-β-catenin2 construct achieved maximal knockdown and was thus used for all downstream experiments.

In order to more fully evaluate the mechanistic basis for the ability of PNS to promote EPC angiogenesis, we therefore next assessed the expression of Wnt/β-catenin signaling pathway proteins in PNS-treated cells. Relative to control and NC group cells, cells in the CTRL + PNS group and NC + PNS groups exhibited significantly elevated nuclear β-catenin levels, while PNS was also able to promote some degree of increased nuclear β-catenin expression in shRNA-β-catenin-treated cells (Fig. 3e, f). In contrast, such treatment had no significant effect on cytoplasmic β-catenin levels in these same cells (Fig. 3e, f). Together, these findings suggest that PNS is capable of driving nuclear β-catenin accumulation while also enhancing overall β-catenin expression when it is downregulated. This thus indicates that a 6.25 mg/L dose of PNS is sufficient to activate Wnt/β-catenin signaling in EPCs.

Our Western blotting results further suggested that PNS treatments were associated with increased expression of DVL and p-GSK-3β in all experimental groups (Fig. 3g, h), while such PNS treatment was also associated with GSK-3β downregulation in all treatment groups (Fig. 3g, h). However, no significant differences were observed within PNS-treated and untreated cell groups, thus suggesting that PNS can activate Wnt/β-catenin signaling via impacting a target gene upstream of β-catenin.

### PNS promotes angiogenesis, tube formation and migration via the Wnt/β-catenin signaling pathway

**Fig. 4:**
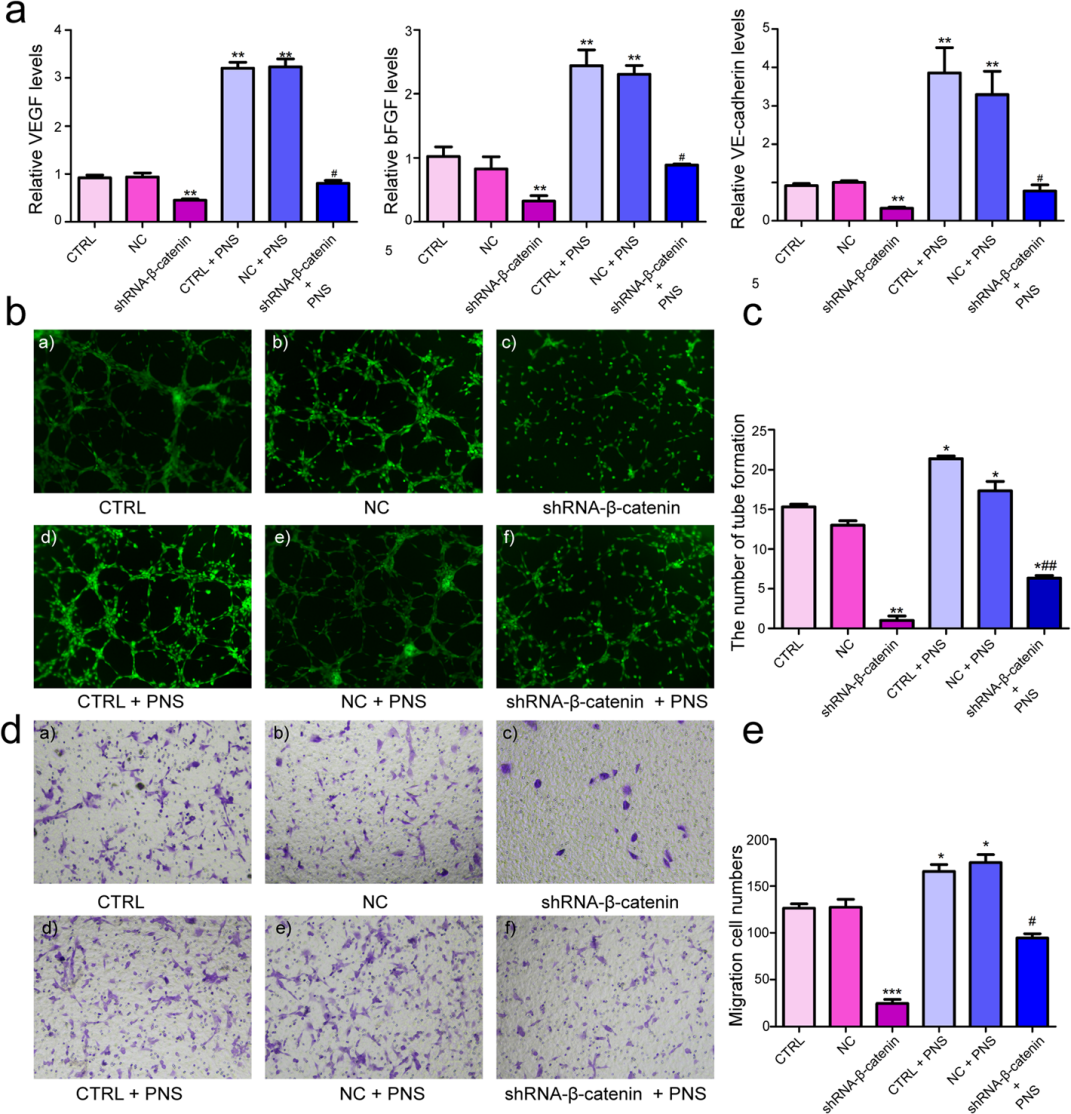
Treatment with PNS rescued tube formation and migration of EPCs transfected by shRNA-β-catenin. **a** EPCs were treated with 6.25 mg/L PNS and incubated for 72 h. The mRNA expression of VEGF, bFGF and VE-cadherin was detected by qRT-PCR. **b**-**c** Silencing of β-catenin inhibits the angiogenesis ability of EPCs, while PNS could reverse the ability in vitro. **p < 0.01, *p < 0.05 v.s. CTRL group, ##*p* < 0.01 v.s. shRNA-β-catenin group. **d**-**e** Migration assay was conducted in EPCs with indicated treatment. The migration cell number was measured at 72 h after the cells had been treated. ***p < 0.001, *p < 0.05 v.s. CTRL group, #*p* < 0.05 v.s. shRNA-β-catenin group

Lastly, to demonstrate the direct role of the Wnt/β-catenin pathway in the PNS promotion of angiogenesis in EPCs, qRT-PCR, WB, the Matrigel tube formation assay, and the migration assay were used to detect EPC angiogenesis after inhibition of β-catenin and treatment with PNS (Fig. 4).

We found that silencing of β-catenin by siRNA treatment abolished both mRNA (Fig. 4a) and protein (Fig. 3g, h) expression of VEGF-A, bFGF and VE-cadherin. Following a 72 h treatment with PNS, we observed that PNS rescued the VEGF**-**A, bFGF and VE-cadherin mRNA levels (Fig. 4a), while only increasing VEGF-A protein levels in shRNA-β-catenin + PNS-treated cells (Fig. 3g h). This suggests that the ability of PNS to drive angiogenesis may be primarily linked to VEGF-A upregulation.

In comparison with the CTRL and NC groups, angiogenesis increased significantly in the PNS + CTRL and PNS + NC groups (Fig. 4b, c; *p* < 0.05) but decreased in the β-catenin inhibitor and β-catenin-inhibitor+PNS groups (Fig. 4b, c; *p* < 0.01). No significant difference in angiogenesis was found between the CTRL and NC groups nor between the CTRL + PNS and NC + PNS groups (Fig. 4b, c; *p* > 0.05). The results also clearly demonstrated that the angiogenetic capability was partially rescued in EPCs treated with PNS after inhibition of β-catenin (Fig. 4b, c; *p* < 0.01). For the migration assay, the results were very similar to the tube formation experiment. The migration of EPCs transfected with the β-catenin inhibitor was significantly decreased compared with the CTRL and NC groups, while treatment with PNS could reverse the effect of the β-catenin inhibitor on migration ability (Fig. 4d-e). These results indicated that angiogenesis of EPCs was benefited by PNS via Wnt/β-catenin signaling pathway.

## Discussion

Bone defects are a great challenge to oral and maxillofacial surgery. DO is a valuable and promising technique that has been widely used for bone regeneration in deformities and defects caused by tumor resection, trauma, congenital abnormality, and infection, amongst others [26]. However, inadequate blood supply may cause delayed bone union, leading to an increased risk of complications and limiting the widespread clinical application of DO. Sufficient vascularity is vital for bone healing, and the rate and range of vascular growth determine the efficiency of osteogenesis [27]. At present, a good solution for ischemic bone defects is lacking. Therefore, the development of novel strategies for accelerating angiogenesis and osteogenesis is urgent and necessary.

Over the last decade, TCM and cell-based therapeutics have gained attention for the treatment of DO in both fundamental and clinical studies. PNS is a TCM treatment that has been employed for centuries in China to treat a variety of diseases and conditions. Many different PNS preparations including injections, powders, and capsules are regularly used [28, 29] and are often prescribed in the context of coronary heart disease owing to their ability to suppress bleeding and to promote circulation. Dong et al. [30] have confirmed that PNS treatment can promote the upregulation of VEGF-A at the mRNA level, thereby suppressing vascular endothelial cell apoptosis and thus potentially aiding in coronary heart disease treatment. Despite significant advances in our knowledge of PNS and its clinical applications in DO [7] and vascular disease [9], the mechanism of PNS in these processes remains unknown. We, therefore, investigated the potential mechanisms.

To the best of our knowledge, this study is the first to investigate the safety and efficiency of PNS by evaluating EPC proliferation, migration, and angiogenesis in vitro. Firstly, the CCK-8 assay, transwell assay, and tube formation assay were performed to confirm the pro-angiogenic effects of PNS on EPCs. Furthermore, qRT-PCR and WB indicated that PNS promotes the expression of VEGF, bFGF and VE-cadherin in EPCs at both the mRNA and the protein level. Based on these results, it is suggested that PNS not only promotes the proliferation, migration and tube formation of EPCs but also enhances the expression of angiogenic-related cytokines and proteins in vitro, making it a prerequisite for DO callus tissue repair and regeneration. Since angiogenesis plays a pivotal role in the process of new bone formation and bone repair during DO, the beneficial effects of PNS on osteogenesis and consolidation during DO shown in our previous study [7] may also enhance angiogenesis. In the present study, we further investigated the mechanism underlying the promotion of angiogenesis by PNS, revealing that this traditional medicinal compound was able to exert its pro-angiogenic effects on these cells via activation of the canonical Wnt/β-catenin pathway. The highly conserved Wnt/β-catenin pathway plays central roles in regulating cellular proliferation, polarity, and differentiation both during embryonic development and in the context of maintaining normal tissue homeostasis [25, 31]. This is the first demonstration of an association between the Wnt signaling pathway and PNS in EPCs. Our results confirmed that PNS could activate the Wnt/β-catenin signaling pathway. Notably, cytoplasmic β-catenin accumulation is followed by its nuclear translocation, wherein it can regulate the transcription of an array of specific target genes [32]. In this study, we found that nuclear translocation of β-catenin was increased by PNS stimulation in comparison with the control group. In order to confirm the functional relevance of PNS-mediated activation of Wnt/β-catenin signaling in these EPCs, we further knocked down β-catenin expression in these cells and demonstrated that PNS treatment was sufficient to overcome the suppression of EPC angiogenesis associated with inhibition of Wnt signaling pathway activity. At baseline, cytosolic β-catenin is phosphorylated by GSK3-β through the action of the APC/Axin/GSK-3β complex and is subsequently degraded [33]. Specific activating stimuli, however, lead to Dvl recruitment and inhibition of this β-catenin degradation complex, thus allowing this transcriptional co-activator to accumulate in the cytoplasm [34]. The context of EPC angiogenesis was associated with upregulated DVL and p-GSK3β expression and with the downregulation of total GSK-3β. These proteins are key regulators acting β-catenin or upstream of β-catenin in the Wnt signaling pathway, thus suggesting that PNS primarily promotes EPC angiogenesis is able to activate Wnt/β-catenin signaling, potentially via either directly impacting β-catenin or by acting on an upstream signaling component in this pathway.

There are several limitations to the present study. For one, PNS is known to be an active mixture of compounds and contains multiple dammarane-type saponins, including ginsenoside-Rh1, ginsenoside Rg1, ginsenoside Re, notoginsenoside R1, ginsenoside Rb1, and ginsenoside Rb1 [35] As such, future studies will be needed to determine which of these compounds are important for EPC angiogenesis and whether a subset of these saponins interact synergistically to drive angiogenic activity.

## Conclusion

**Fig. 5:**
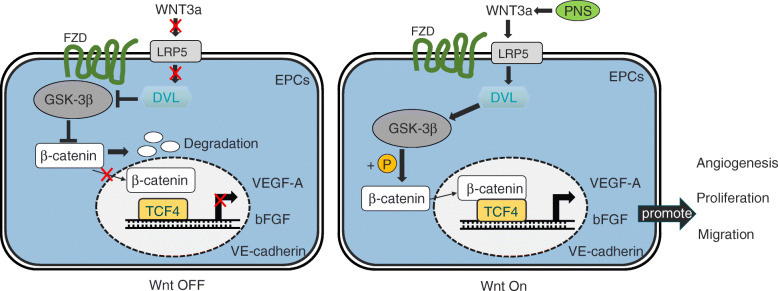
Schematic of the detailed mechanisms involved in PNS-mediated angiogenesis of EPCs. Left: In the absence of a Wnt signal, β-catenin is degraded by a complex of proteins including GSK-3β. DVL is required for activating the pathway as well. Right: PNS could promote WNT3a and bind of LRP5 to induces the association of DVL with phosphorylated GSK-3β. The destruction complex falls apart, and β-catenin is stabilized, subsequently binding TCF4 in the nucleus to upregulate angiogenesis related cytokines and promote EPCs angiogenesis, proliferation and migration

In summary, our results reveal that 6.25 mg/L PNS promotes EPC angiogenesis via activation of the Wnt/β-catenin pathway (Fig. 5). Together, these results offer novel insights into the mechanistic basis for the pro-angiogenic properties of PNS while providing a theoretical foundation for future studies of the potential clinical application of this traditional medicinal compound.

## Data Availability

The supporting materials used in this study are contained within the article.

## Abbreviations

bFGF: Basic fibroblast growth factor
CTRL: Control
CCK-8: CCK-8 Cell Counting Kit-8
DO: Distraction osteogenesis
EPCs: Endothelial progenitor cells
GSK-3β: Glycogen synthase kinase-3b
NC: Negative control
PNS: Panax notoginseng saponins
p-GSK3β: Phosphorylated glycogen synthase kinase-3b
PBS: Phosphate-buffered saline
PMSF: Phenylmethanesulfonyl fluoride
PVDF: Polyvinylidene fluoride
PFA: Paraformaldehyde
RIPA: Radio immunoprecipitation assay
SDS: Sodium dodecyl sulfate
TCM: Traditional Chinese medicine
VEGF: Vascular endothelial growth factor
VE-cadherin: Vascular endothelial cadherin
